# Interleukins-27 Aggravates Liver Injury by Impairing the Antimicrobial Response of Macrophages via the Promotion of Mitochondrial Dysfunction in the Context of Sepsis

**DOI:** 10.1155/mi/6608718

**Published:** 2025-02-26

**Authors:** Yuehua You, Yuyan Li, Lin Ye, Fang Xu, Jing Fan

**Affiliations:** ^1^Department of Critical Care Medicine, The First Affiliated Hospital of Chongqing Medical University, Chongqing, China; ^2^The Chongqing Key Laboratory of Translational Medicine in Major Metabolic Diseases, The First Affiliated Hospital of Chongqing Medical University, Chongqing, China; ^3^Department of Critical Care Medicine, The First People's Hospital of Chongqing High-tech Zone, Chongqing, China; ^4^Laboratory Research Center, The First Affiliated Hospital of Chongqing Medical University, Chongqing, China

**Keywords:** AHI, IL-27, macrophages, mitochondrial dysfunction, sepsis

## Abstract

**Background and Aims:** Plasma interleukin (IL)-27 is an important mediator of acute hepatic injury (AHI) associated with sepsis. Mitochondria contribute to the proper regulation of macrophage phagocytosis. In this study, we investigated the effect of IL-27 on mitochondrial function and the antimicrobial response of macrophages in sepsis-associated AHI.

**Methods:** Wild-type (WT) and IL-27 receptor WSX-1 deficient (IL-27R^−/−^) mice underwent cecal ligation and puncture (CLP). The severity of hepatic injury, inflammatory cytokine levels, hepatic pyroptosis, and bacterial load in the liver and blood were assessed 24 h after CLP. In vitro, RAW264.7 cells and peritoneal macrophages were treated with lipopolysaccharide (LPS) and/or IL-27. The phagocytosis and killing functions of macrophages were detected. Mitochondrial function and mitophagy were detected using western blot, glutathione (GSH)/malondialdehyde (MDA) content measurement, fluorescence staining, and JC-1 staining in vivo and in vitro. After treatment with nicotinamide mononucleotide (NMN, NAD + precursor), a pharmacologic agent that improves mitochondrial function, the inflammatory response, hepatic injury, and hepatic pyroptosis were assessed.

**Results:** IL-27R^−/−^ mice exhibited a marked reduction in hepatic injury, pyroptosis (based on cleaved GSDMD and cleaved Caspases 1 protein levels), and systemic inflammation (based on serum IL-6, IL-10, and TNF-*α* levels) compared to WT mice following CLP. After CLP, mice lacking IL-27R displayed significantly higher bacterial clearance and greater local infection control. Subsequent studies demonstrated that IL-27 directly impaired the LPS-induced bacterial phagocytosis, killing capacity, and mitochondrial function of macrophages. Finally, enhanced mitochondrial function using NMN in vivo significantly alleviated pathological liver injury and inflammation.

**Conclusions:** These findings indicated that IL-27 impairs the bacterial phagocytosis capacity of macrophages by aggravating mitochondrial dysfunction to aggravate AHI during sepsis.

## 1. Introduction

Sepsis, defined as a disordered and exaggerated systemic inflammatory response to infection, is the main cause of death in intensive care units (ICUs) [1]. Multiorgan dysfunction triggered by an imbalanced inflammatory response is the direct reason for the high mortality rate associated with sepsis [2]. Acute hepatic injury (AHI) is an independent predictor of poor prognosis in patients with sepsis [3]. Although the exact pathophysiological mechanism of sepsis-associated AHI is not yet clear, the effect of an uncontrolled inflammatory cascade response induced by pathogens on the occurrence of AHI has been widely recognized [4–6].

Macrophages are the main immune cell group in the liver, and they are crucial for the liver to participate in immune and inflammatory regulation [7]. They can release excessive quantities of pro- and anti-inflammatory cytokines that disrupt the liver immune balance during sepsis and promote local and systemic inflammatory reactions, thereby lead to AHI [8]. However, certain cytokines can act on macrophages to regulate their function [9]. Thus, the interactions between liver macrophages and cytokines form a complex inflammatory network, resulting in AHI, unbalanced organ crosstalk, and poor outcome [10].

Interleukin (IL)-27, which is composed of Epstein–Barr virus-induced gene 3 (EBi3) and p28 subunits, is a member of the IL-6/IL-12 family [11]. It can be synthesized by macrophages, and it influences macrophage differentiation [12, 13]. IL-27 is a pleiotropic cytokine that exerts different effects in different inflammatory environments [14]. Our previous studies found that IL-27 levels are elevated in sepsis-associated AHI in humans and in a mouse model of sepsis and that IL-27 plays a role in promoting macrophage M1 polarization and increasing IL-6 and TNF-*α* levels [15]. However, the complex mechanism through which IL-27 regulates macrophage function in the inflammatory microenvironment of the liver during sepsis remains unclear.

Mitochondria play important roles in regulating immune cell function and shaping and modulating the immune system's response to infections [16]. Mitochondrial dysfunction affects cellular metabolism and the release of damage-associated molecular patterns (DAMPs), thereby activating inflammatory macrophages [17]. In contrast, mitochondria affect the phagocytic function of macrophages via the production of reactive oxygen species (ROS) [18]. IL-27 is a potent regulator of ROS induction in macrophages [19]. However, in the inflammatory process of sepsis-associated AHI, it is unclear whether mitochondrial function is also a target of IL-27 to affect the degree of liver injury by regulating macrophage activation. Therefore, we established a mouse model of sepsis-associated AHI and a macrophage model activated by inflammation to explore the mechanisms through which IL-27 regulates mitochondrial function to active the macrophages and, thereby, influence liver injury.

## 2. Materials and Methods

### 2.1. Animals

Male C57BL/6J mice were obtained from Laboratory Animal Center of Chongqing Medical University (Chongqing, China). The mice were maintained on a 12-h light/dark cycle and had unrestricted access to food and water. Wild-type (WT) and IL-27R^−/−^ mice were subjected to cecal ligation and puncture (CLP). Briefly, the mice were anesthetized, and a 1 cm cut was made down the center of the abdomen. After ligating the appendix, the needle was punctured. After restoration to the normal position, the wound was sutured after an intraperitoneal injection of 1 mL of saline. The sham group received the same treatment, except that the appendix was not ligated and punctured. All research procedures were approved by the Institutional Animal Care and Use Committees at Chongqing Medical University (reference number IACUC-CQMU-2024-0123).

### 2.2. Bacterial Load

Animals were sacrificed under anesthesia, and after general disinfection with 75% alcohol, blood was extracted by extracorporeal cardiac puncture into a sterile anticoagulation tube. In a sterile environment, the abdomen was incised, and the liver was extracted and placed in a clean Eppendorf tube. Subsequently, the livers were homogenized in 1 mL of sterile ice-cold phosphate-buffered saline (PBS). The blood and liver homogenization solution was diluted by factors of 10, 100, and 1000. The solution was then cultured on Columbia Blood Agar and incubated at 37°C overnight. Bacterial colony-forming units (CFUs) were counted to calculate bacterial loads in the blood and liver samples of each group.

### 2.3. Macrophage Phagocytosis and Killing Experiment

The mice were intraperitoneally injected with sterile paraffin oil. After 7 days, they were anesthetized, and the peritoneal macrophages were collected by rinsing the peritoneal cavity with PBS. Cells were counted and seeded in 24-well plates at a density of 5 × 10^5^ cells/well. The macrophages were treated with lipopolysaccharide (LPS) and/or IL-27 after 36 h of incubation. They were then infected with standard *P. aeruginosa* strains (5 × 10^7^ CFU per well) and placed on a shaker at 37°C for 30 min. After rinsing three times with PBS, gentamicin (200 μg/mL, Solarbio, Beijing, China) was added for 15 min at ambient temperature to eliminate external bacteria. Following another wash with PBS, 200 μL of double-distilled (dd) H_2_O was added to each well, and the phagocytosis plate was incubated for 20 min at ambient temperature to liberate intracellular bacteria. To prepare the killing plate, 1 mL of Dulbecco's modified Eagle medium (DMEM; Gibco, Waltham, MA, USA) was added to each well, and the plate was incubated for 2 h. After three times with PBS, the cells were lysed in 200 μL of ddH_2_O at room temperature for 20 min. After diluting the lysed bacteria from the phagocytosis and killing plates by factors of 10, 100, and 1000, they were inoculated onto blood plates and incubated at 37°C overnight. The number of colonies was counted to determine the phagocytic and killing capabilities of the macrophages in each group.

### 2.4. Cell Culture

RAW264.7 cells were grown in DMEM containing 10% fetal bovine serum (FBS) and exposed to LPS (100 ng/mL; Sigma–Aldrich, St Louis, MO, USA) and/or IL-27 (50 ng/mL; ProSpec, Rehovot, Israel) for 36 h when they reached 70%–80% confluences.

### 2.5. Drug Intervention

Recombinant IL-27 was injected intraperitoneally at a dose of 50 μg/kg 2 h before CLP was operated, while nicotinamide mononucleotide (NMN) was injected intraperitoneally 30 min after the *procedure* at a dose of 500 mg/kg [20]. RAW264.7 cells were treated with LPS, IL-27, and/or NMN (500 μM).

### 2.6. Quantitative Reverse Transcription-Polymerase Chain Reaction (PCR)

RNAiso plus (Takara, Kusatsu, Japan) was used to extract total RNA from RAW264.7 cells, after which cDNA was synthesized using a PrimeScript RT reagent kit (Takara). Subsequently, reverse transcription-PCR was performed with SYBR Green PCR Master Mix (Takara). Gene expression levels were normalized to *β*-actin expression levels using the 2−*ΔΔ*Ct method.

### 2.7. Intracellular Calcium (Ca^*2+*^) Concentration Measurement

RAW264.7 cells were collected in a centrifuge tube and stained with Fluo-4 staining solution (Beyotime-Biotechnology, Shanghai, China) at 37°C in the dark for 30 min. After washing with PBS, green fluorescence was detected using flow cytometry.

### 2.8. Histology and Staining

Livers were fixed with 4% paraformaldehyde, embedded in paraffin, and stained with hematoxylin and eosin. Hepatic damage levels were assessed using the histological activity index (HAI), which includes measurements for portal necrosis (0–10), intralobular hepatocyte degeneration and focal necrosis (0–4), portal inflammation (0–4), and fibrosis (0–4).

### 2.9. Serum Alanine Transaminase (ALT) and Aspartate Transaminase (AST) Levels Measurement

Blood samples were centrifuged at 4°C for 10 min to collect serum. The serum levels of ALT and AST were determined using an ALT/AST assay kit (Solarbio).

### 2.10. Intracellular ROS Measurements and MitoTracker Staining

ROS levels in treated RAW264.7 cells were measured using an ROS staining kit (Beyotime Biotechnology). Briefly, RAW264.7 cells were exposed to LPS and/or IL-27 and were then exposed to a 5 μM ROS probe for 20 min at 37°C. The cells were exposed to 100 nM MitoTracker probes (Invitrogen, Carlsbad, CA, USA) and incubated for 20 min at 37°C. After washing with PBS, fluorescence was analyzed using flow cytometry. Data analysis was performed using FlowJo software V10 (FlowJo LLC, Ashland, OR, USA).

### 2.11. Adenosine Triphosphate (ATP) Content

ATP levels in RAW264.7 cells were measured using an ATP detection kit (Beyotime Biotechnology). After lysing the cells in 200 μL of lysis buffer, they were centrifuged at 12,000 × *g* for 5 min at 4°C, and the resulting supernatant was collected to quantify the ATP content. After adding 100 μL of detection-working solution into a black 96-well plate and incubating for 5 min, 40 μL of the cell supernatant was added, and luminescence was measured using a SpectraMax L (Molecular Devices, Shanghai, China) instrument.

### 2.12. Measurement of Mitochondrial Membrane Potential (*Δψ*m)

Δ*ψm* provides a valuable indicator of mitochondrial functional status and is usually evaluated using the dye JC-1 [21]. JC-1 selectively enters mitochondria, and its fluorescence characteristics (red/green fluorescence) change with changes in mitochondrial *Δψ*m. RAW264.7 cells were cultured in 12-well dishes and exposed to LPS and/or IL-27 for 36 h. The cells were then stained with JC-1 (Beyotime-Biotechnology) at 37°C for 20 min. After washing with dyeing buffer, green (J-monomer) and red (J-aggregates) fluorescence were observed under a ZEISS DM4B fluorescence microscope (ZEISS, Germany).

### 2.13. Measurement of Superoxide Dismutase (SOD) Activity, Glutathione (GSH) Levels, and Malondialdehyde (MDA) Levels

SOD activity (Beyotime Biotechnology), GSH levels (Nanjing Jiancheng Reagents, Nanjing, China), and MDA levels (Nanjing Jiancheng Reagents) were measured in liver tissues and RAW264.7 cells using a commercial assay kit.

### 2.14. Protein Extraction and Western Blotting

Mouse livers and RAW264.7 cells were lysed using RIPA with phenylmethanesulfonyl fluoride (PMSF) and phosphatase inhibitors (Beyotime Biotechnology). Protein specimens (30 μg) were separated on 12% sodium dodecyl sulfate-polyacrylamide gels and transferred to a polyvinylidene fluoride (PVDF) membrane (Millipore, USA). Following blocking with 5% nonfat milk, the blots were incubated with primary antibodies targeting OXPHOS (20/30/40/48/55 kDa; Abcam, Cambridge, UK;1/500), *p*-Parkin (50 kDa; Affinity Biosciences, Cincinnati, OH, USA; 1/1000), Parkin (52 KDa; ABclonal, Wuhan, China; 1/1000), PINK1 (63 kDa; ABclonal, 1/1000), LC3A/B (14/16 kDa; Cell Signaling Technology, Danvers, MA, USA; 1/1000), SOD1 (15 kDa; Proteintech, San Diego, CA, USA; 1/1000), GSDMD (30/50 kDa; Abcam, 1/1000), Caspase 1 (10/48 kDa, Cell Signaling Technology, 1/1000), TOM20 (15 kDa; Proteintech, 1/1000) and ACTB (42 kDa, ABclonal, 1/3000). Following incubation with an HRP-conjugated goat anti-rabbit/mouse secondary antibody, the blots were exposed using the Fusion FX Spectra system (Vilber Lourmat, Eberhardzell, Germany).

### 2.15. Enzyme-Linked Immunosorbent Assay (ELISA)

Serum levels of TNF-*α*, IL-6, IL-1*β*, and IL-10 were measured using ELISA kits (4A Biotech, Beijing, China) following the manufacturer's guidelines. Absorbance was measured at 450 nm, and serum inflammatory factor concentrations were calculated from the standard curve.

### 2.16. Immunofluorescence Staining

RAW264.7 cells were treated with 4% PFA for 15 min and then permeabilized with 0.5% Triton X-100. Following the block with goat serum at room temperature for 45 min, the cells were incubated overnight at 4°C with primary antibodies targeting TOM20 and LC3 and then with FITC- or CY3-conjugated secondary antibodies at room temperature for 30 min (1:200, Affinity Biosciences). After three washes with PBS with Tween 20, the cells were stained with DAPI, and the images were observed using a confocal fluorescence microscope (Zeiss DM4B).

### 2.17. Mitochondrial Protein Isolation Assay

Mitochondrial proteins were isolated from RAW264.7 cells using the Cell Mitochondrial Isolation Kit (Beyotime Biotechnology). Briefly, RAW264.7 cells were homogenized in 1 mL of mitochondrial isolation reagent containing 1% PMSF. The supernatant containing the mitochondrial fraction was collected after centrifugation at 600 × *g* for 10 min at 4°C. The supernatant was centrifuged at 11,000 × *g* for 10 min at 4°C to collect mitochondria from the cells.

### 2.18. Immunohistochemical Staining

Liver tissue samples from mice were fixed in paraffin and then treated with anti-IL-1*β* antibody (Abcam) following peroxidase inactivation. A secondary antibody was then added, followed by diaminobenzidine (DAB), and images were captured using a fluorescence microscope (Zeiss DM4B).

### 2.19. Coculture

RAW264.7 cells were treated with LPS, IL-27, and/or NMN for 36 h. The culture supernatant was then added to HepG2 cells for 24 h. Pyroptosis of HepG2 cells was detected by western blotting.

### 2.20. Statistical Analysis

GraphPad Prism 10.0 was used for statistical analysis. Data are expressed as mean + standard error (SE). One-way ANOVA, followed by Tukey's test, was used to analyze the data.

## 3. Results

### 3.1. Knockout of IL-27 Receptor Ameliorated Hepatic Injury in CLP Mice

**Figure 1:**
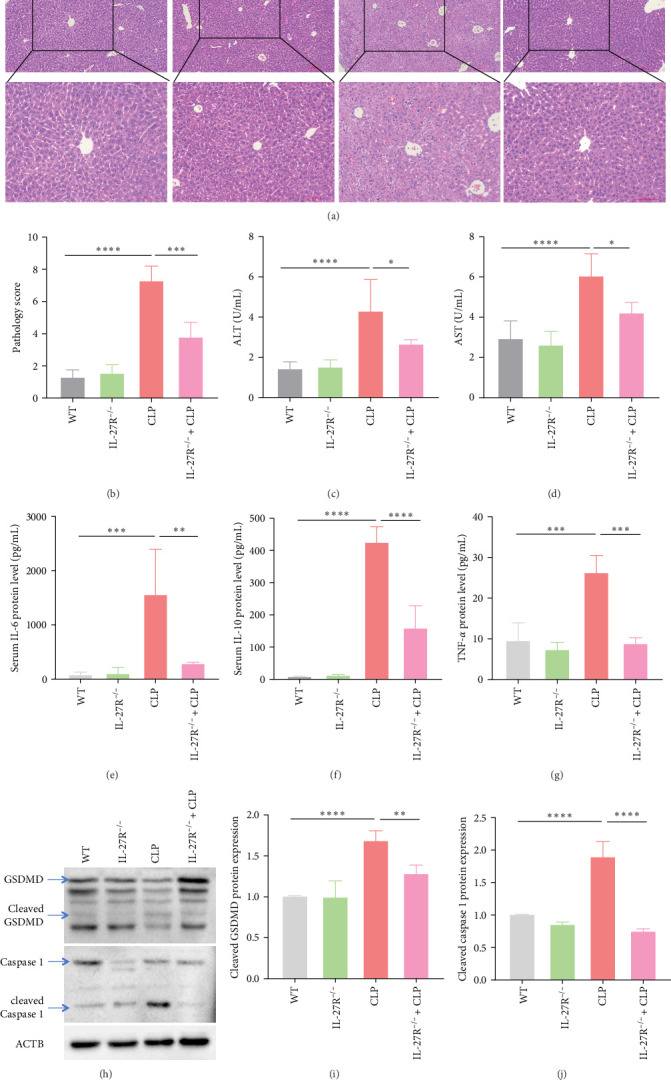
Knockout of IL-27 receptor ameliorated hepatic injury in CLP mice. (A) Mouse hepatic pathological injury was detected by H&E staining (*n* = 4). (B) The pathology score determined from H&E staining (*n* = 4; WT vs. CLP: *p* < 0.0001, CLP vs. IL-27R^−/−^+CLP: *p*=0.0002). (C) Serum ALT level (*n* = 5; WT vs. CLP: *p* < 0.0001, CLP vs. IL-27R^−/−^+CLP: *p*=0.0147). (D) Serum AST level (*n* = 5; WT vs. CLP: *p* < 0.0001, CLP vs. IL-27R^−/−^+CLP: *p*=0.0116). (E) The concentration of inflammatory cytokines IL-6 in mouse serum was measured using ELISA (*n* = 5; WT vs. CLP: *p*=0.0005, CLP vs. IL-27R^−/−^+CLP: *p*=0.0033). (F) The concentration of inflammatory cytokines IL-10 in mouse serum was measured using ELISA (*n* = 5; WT vs. CLP: *p* < 0.0001, CLP vs. IL-27R^−/−^+CLP: *p* < 0.0001). (G) The concentration of inflammatory cytokines TNF-*α* in mouse serum was measured using ELISA (*n* = 4; WT vs. CLP: *p*=0.0002, CLP vs. IL-27R^−/−^+CLP: *p*=0.0001). (H) Western blotting was used to measure the expression levels of GSDMD and Caspase 1 protein levels in liver tissue (*n* = 4). (I) Statistical analysis of relative GSDMD protein expression levels (*n* = 4; WT vs. CLP: *p* < 0.0001, CLP vs. IL-27R^−/−^+CLP: *p*=0.0051). (J) Statistical analysis of relative Caspase 1 protein expression levels (*n* = 4; WT vs. CLP: *p* < 0.0001, CLP vs. IL-27R^−/−^+CLP: *p* < 0.0001). All images were randomly obtained from 40× and 100× visual fields. *⁣*^*∗*^*p* < 0.05, *⁣*^*∗∗*^*p* < 0.01, *⁣*^*∗∗∗*^*p* < 0.001, *⁣*^*∗∗∗∗*^*p* < 0.0001. ALT, alanine transaminase; AST, aspartate transaminase; CLP, cecal ligation and puncture; ELISA, enzyme-linked immunosorbent assay; IL, interleukin; WT, wild-type.

To elucidate the role of IL-27 in hepatic injury associated with sepsis, WT and IL-27R^−/−^ mice were subjected to CLP, and the degree of liver injury was evaluated by histopathological methods (H&E). As shown in Figure 1A,B (*n* = 4), the liver injury score was significantly higher in the CLP group than in the control group. However, compared with WT mice, IL-27R^−/−^ mice showed significantly ameliorated hepatic injury after CLP. Further examination of serum ALT and AST levels revealed higher circulating AST and ALT levels in the CLP group, and IL-27R knockout reduced AST and ALT levels (Figure 1C,D; *n* = 5). In addition, the levels of serum inflammatory factors IL-6, IL-10, and TNF-*α* were also significantly reduced in mice subjected to CLP after IL-27 receptor knockdown (Figure 1E–G; E and F: *n* = 5; G: *n* = 4). In addition, we assessed hepatic pyroptosis by measuring GSDMD and Caspases 1 protein levels. As depicted in Figure 1H–J (*n* = 4), hepatic cleaved GSDMD and cleaved Caspases 1 protein levels were significantly higher in the CLP group than the control group, and IL-27R knockout significantly ameliorated the hepatic pyroptosis in the livers of CLP mice.

### 3.2. IL-27 Impaired the Phagocytosis and Killing Function of Macrophages

**Figure 2:**
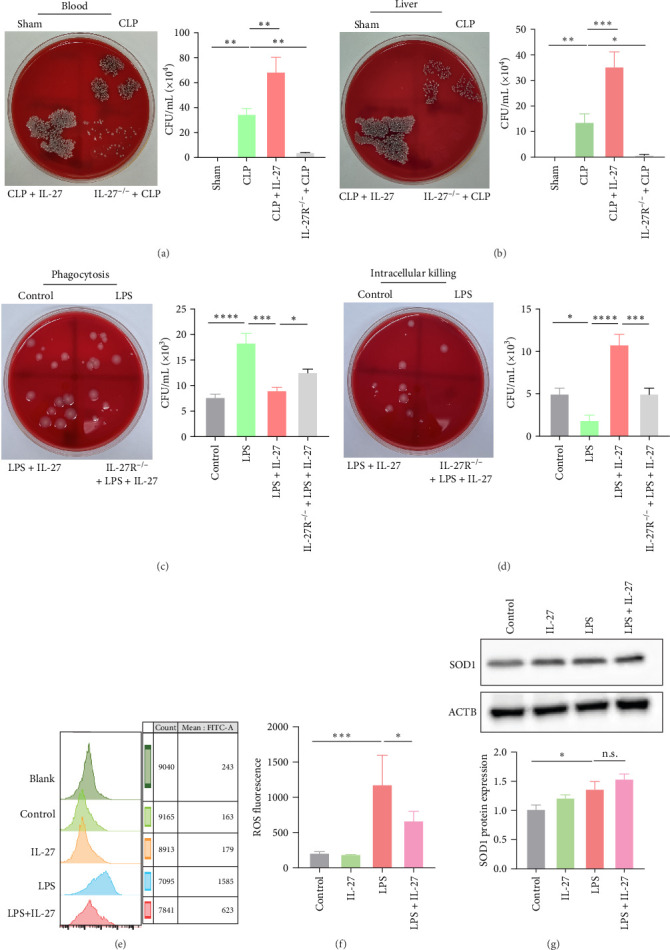
IL-27 impaired the phagocytosis and killing function of macrophages. (A) The bacterial load in the blood of WT and IL-27R^−/−^ mice 24 h after CLP (*n* = 3; Sham vs. CLP *p*=0.0011, CLP vs. CLP + IL-27 *p*=0.0011, CLP vs. IL-27R^−/−^+CLP *p*=0.0023). (B) The bacterial load in the liver of WT and IL-27R^−/−^ mice 24 h after CLP (*n* = 3; Sham vs. CLP *p*=0.0079, CLP vs. CLP + IL-27 *p*=0.0003, CLP vs. IL-27R^−/−^+CLP *p*=0.0100). (C) Changes in the phagocytosis function of macrophages after treatment with LPS and/or IL-27 (*n* = 3; Control vs. LPS *p* < 0.0001, LPS vs. LPS + IL-27 *p*=0.0003, LPS + IL-27 vs. IL27R^−/−^+LPS + IL-27 *p*=0.0295). (D) Changes in the killing function of macrophages after treatment with LPS and/or IL-27 (*n* = 3; Control vs. LPS *p*=0.0145, LPS vs. LPS + IL-27 *p*  < 0.0001, LPS + IL-27 vs. IL27R^−/−^+LPS + IL-27 *p*=0.0003). (E) ROS levels in RAW264.7 cells were detected by flow cytometry (*n* = 4). (F) Quantitative analysis of ROS fluorescence levels (*n* = 4; Control vs. LPS *p*=0.0003, LPS vs. LPS + IL-27 *p*=0.0369). (G) Western blotting was used to analyze the expression levels of SOD1 protein in RAW264.7 cells (*n* = 3; Control vs. LPS *p*=0.0123, LPS vs. LPS + IL-27 *p*=0.2328). *⁣*^*∗*^*p* < 0.05, *⁣*^*∗∗*^*p* < 0.01, *⁣*^*∗∗∗*^*p* < 0.001, *⁣*^*∗∗∗∗*^*p* < 0.0001. CLP, cecal ligation and puncture; IL, interleukin; LPS, lipopolysaccharide; ROS, reactive oxygen species; SOD, superoxide dismutase; WT, wild-type.

We next observed the bacterial load in the blood and liver of WT and IL-27R^−/−^ mice 24 h after CLP. The bacterial load in both the blood and liver significantly increased after CLP and further increased after intervention with IL-27 (Figure 2A,B; *n* = 3). However, the bacterial loads were significantly lower in IL-27R^−/−^ mice than in WT mice after CLP (Figure 2A,B). Next, we extracted peritoneal macrophages to observe the effect of IL-27 on phagocytosis and the killing functions of macrophages. As shown in Figure 2C,D (*n* = 3), IL-27 significantly impaired LPS-induced phagocytosis and killing of macrophages. However, IL-27R knockout markedly reversed this effect of IL-27. Since ROS levels are associated with the killing function of macrophages, we next explored whether IL-27 treatment altered LPS-induced changes in ROS levels in RAW264.7 cells. Flow cytometry showed that LPS treatment increased ROS levels; however, this effect was reversed by IL-27 (Figure 2E,F; *n* = 4). Moreover, SOD protein levels were further increased after IL-27 treatment compared to those in the LPS group, although the difference was not statistically significant (Figure 2G; *n* = 3).

### 3.3. IL-27 Exacerbated LPS-Induced Mitochondrial Dysfunction in Macrophages

**Figure 3:**
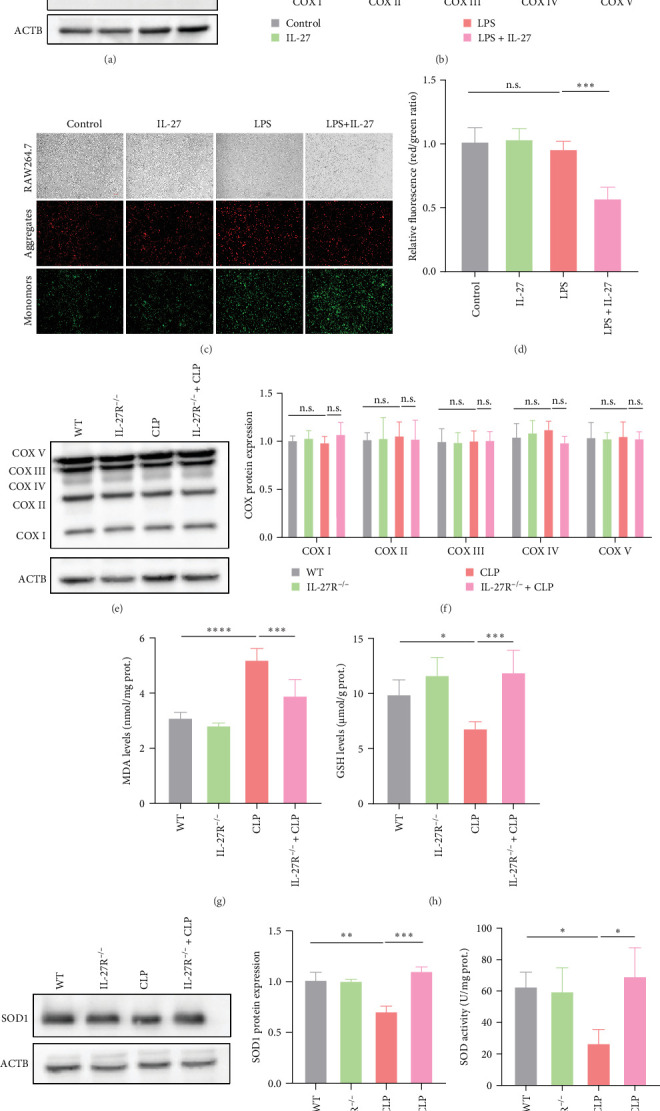
IL-27 exacerbated LPS-induced mitochondrial dysfunction in macrophages. (A) COX protein levels in RAW264.7 cells were detected by western blotting (*n* = 4). (B) Relative statistical analysis of COX protein expression (*n* = 4; COX I: Control vs. LPS *p* < 0.0001, LPS vs. LPS + IL-27 *p*=0.0071; COX II: Control vs. LPS *p* < 0.0001, LPS vs. LPS + IL-27 *p*=0.0364; COX III: Control vs. LPS *p*=0.0102, LPS vs. LPS + IL-27 *p*=0.5266; COX IV: Control vs. LPS *p*  < 0.0001, LPS vs. LPS + IL-27 *p*=0.2252; COX V: Control vs. LPS *p*=0.9998, LPS vs. LPS + IL-27 *p*  > 0.9999). (C) JC-1 staining was used to detect mitochondrial membrane potential (*n* = 4). (D) Relative statistical analysis of JC-1 fluorescence (red/green ratio) (*n* = 4; Control vs. LPS *p*=0.8026, LPS vs. LPS + IL-27 *p*=0.0005). (E) Western blotting was used to detect hepatic COX protein expression (*n* = 4). (F) Statistical analysis of relative hepatic COX protein expression levels (*n* = 4; COX I: WT vs. CLP *p*=0.9059, CLP vs. IL-27R^−/−^+CLP *p*=0.8150; COX II: WT vs. CLP *p*=0.9993, CLP vs. IL-27R^−/−^+CLP *p*=0.9662; COX III: WT vs. CLP *p*=0.9725, CLP vs. IL-27R^−/−^+CLP *p*=0.9890; COX IV: WT vs. CLP *p*=0.7808, CLP vs. IL-27R^−/−^+CLP *p*=0.3914; COX V: WT vs. CLP *p*=0.9983, CLP vs. IL-27R^−/−^+CLP *p*  > 0.9999). (G and H) The levels of hepatic MDA (*n* = 5; WT vs. CLP *p*  < 0.0001, CLP vs. IL-27R^−/−^+CLP *p*=0.0007) and GSH (*n* = 5; WT vs. CLP *p*=0.0294, CLP vs. IL-27R^−/−^+CLP *p*=0.0005). (I) Hepatic SOD1 protein expression was detected by western blotting (*n* = 3; WT vs. CLP *p*=0.0012, CLP vs. IL-27R^−/−^+CLP *p*=0.0002). (J) Changes in hepatic SOD activity in WT and IL-27R^−/−^ mice after CLP (*n* = 4; WT vs. CLP *p*=0.0470, CLP vs. IL-27R^−/−^+CLP *p*=0.0151). *⁣*^*∗*^*p* < 0.05, *⁣*^*∗∗*^*p* < 0.01, *⁣*^*∗∗∗*^*p* < 0.001, *⁣*^*∗∗∗∗*^*p* < 0.0001. GSH, glutathione; IL, interleukin; LPS, lipopolysaccharide; MDA, malondialdehyde; SOD, superoxide dismutase; WT, wild-type.

To determine whether IL-27 impaired mitochondrial function in RAW264.7 cells, we examined the expression of COX proteins, which are mitochondrial electron transport chain complexes. After LPS treatment, a marked reduction in COX I, -II, -III, and -IV expression levels was observed, whereas the COX V expression level was not significantly affected (Figure 3A,B; *n* = 4). IL-27 further reduced COX I and COX II expression levels, although no significant changes in COX III, IV, and V protein levels were observed (Figure 3A,B). Further studies were conducted on RAW264.7 cells treated with LPS or/and IL-27 to determine the mitochondrial membrane potential. Fluorescence images of JC-1-stained cells indicated that IL-27 impaired LPS-induced mitochondrial membrane potential reduction (Figure 3C,D; *n* = 4). To further examine the effect of IL-27 on mitochondrial function in the liver of mice subjected to CLP, we examined COX expression. As depicted in Figure 3E,F (*n* = 4), no alterations in hepatic COX protein levels were observed in mice subjected to CLP. In addition, livers of the CLP group showed elevated levels of MDA levels (Figure 3G; *n* = 5) and reduced GSH (Figure 3H; *n* = 5), and SOD1 protein levels (Figure 3I; *n* = 3) and SOD activity (Figure 3J; *n* = 4). However, IL-27R^−/−^ mice showed significantly restored hepatic MDA, GSH, and SOD1 protein levels and SOD activity compared with those in the control group (Figure 3G–J).

### 3.4. IL-27 Increased LPS-Induced Mitophagy in Macrophages

**Figure 4:**
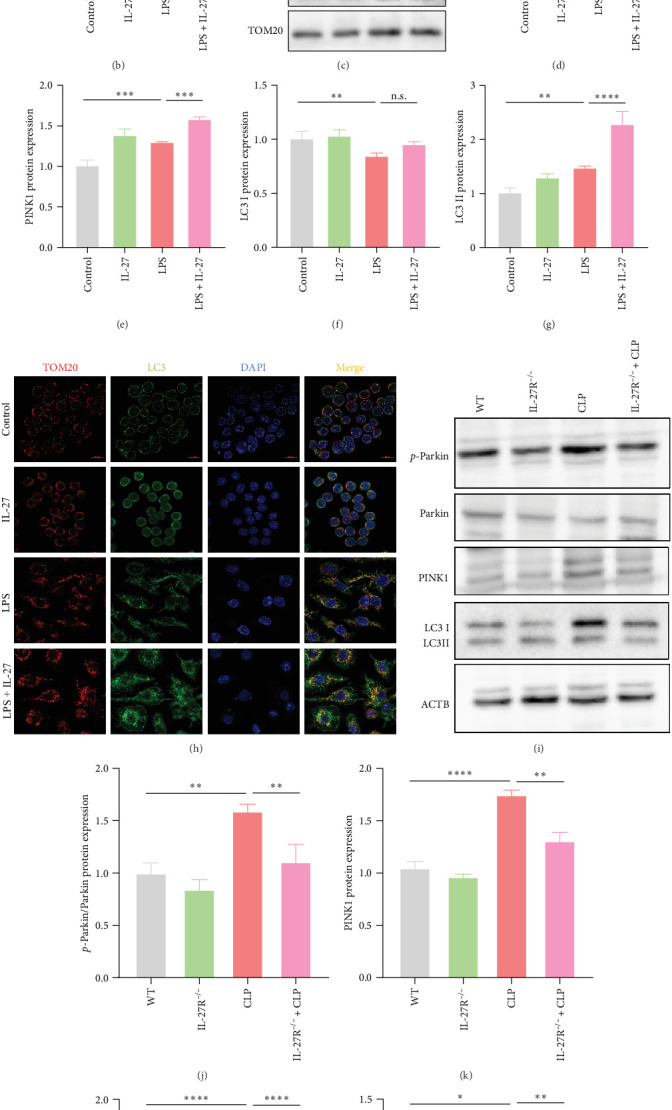
IL-27 increased LPS-induced mitophagy in macrophages. (A) The number of mitochondria was detected using MitoTracker (*n* = 4). (B) MitoTracker red fluorescence quantification analysis (*n* = 4; Control vs. LPS *p*  < 0.0001, LPS vs. LPS + IL-27 *p*=0.0004). (C) Western blotting was used to detect the mitophagy-related protein expression (*p*-Parkin, Parkin, PINK1, and LC3) (*n* = 4). (D–G) Relative statistical analysis of *p*-Parkin/Parkin (*n* = 4; Control vs. LPS*p*  < 0.0001, LPS vs. LPS + IL-27 *p*=0.1192), PINK1 (*n* = 4; Control vs. LPS *p*=0.0001, LPS vs. LPS + IL-27 *p*=0.0002), LC3 I (*n* = 4; Control vs. LPS *p*=0.0057, LPS vs. LPS + IL-27 *p*=0.0649) and LC3 II (*n* = 4; Control vs. LPS *p*=0.0038, LPS vs. LPS + IL-27 *p*  < 0.0001) protein expression. (H) TOM20 and LC3 immunofluorescence staining were used to assess mitophagy (*n* = 4). (I) Western blotting was used to detect the hepatic *p*-Parkin/Parkin, PINK1, LC3 I, and LC3 II (*n* = 3) protein expression. (J–M) Statistical analysis of relative hepatic *p*-Parkin/Parkin (*n* = 3; WT vs. CLP *p*=0.0018, CLP vs. IL-27R^−/−^+CLP *p*=0.0065), PINK1 (*n* = 3; WT vs. CLP *p*  < 0.0001, CLP vs. IL-27R^−/−^+CLP *p*=0.0003), LC3 I (*n* = 3; WT vs. CLP *p*  < 0.0001, CLP vs. IL-27R^−/−^+CLP *p*  < 0.0001) and LC3 II (*n* = 3; WT vs. CLP *p*=0.0154, CLP vs. IL-27R^−/−^+CLP *p*=0.0077) protein expression. *⁣*^*∗*^*p* < 0.05, *⁣*^*∗∗*^*p* < 0.01, *⁣*^*∗∗∗*^*p* < 0.001, *⁣*^*∗∗∗∗*^*p* < 0.0001. CLP, cecal ligation and puncture; IL, interleukin; LPS, lipopolysaccharide; WT, wild-type.

In addition to mitochondrial function, the number of mitochondria also affects ATP production. MitoTraker staining was used to elucidate the effect of IL-27 on the number of mitochondria. The results showed that LPS significantly increased the number of mitochondria in RAW264.7 cells, and of note, the number of mitochondria was further increased in the IL-27+LPS group (Figure 4A,B; *n* = 4)). As a key link affecting the number of mitochondria, we examined the expression of proteins related to the classical mitophagy pathway. Western blotting showed that LPS upregulated the protein levels of *p*-Parkin, PINK1, and LC3II in the mitochondria of macrophages, and the levels of these proteins were further increased by IL-27 treatment (Figure 4C–G; *n* = 4). Western blotting showed that LPS upregulated the protein levels of *p*-Parkin, PINK1, and LC3II in the mitochondria of macrophages, and the levels of these proteins were further increased by IL-27 treatment (Figure 4C–G). Immunofluorescence staining for TOM20 and LC3 also showed that IL-27 further upregulated LPS-induced mitophagy (Figure 4H; *n* = 4). The effect of IL-27 on liver mitophagy in mice subjected to CLP was further observed in IL-27R^−/−^ mice. As illustrated in Figure 4I–M (*n* = 3), IL-27R knockout decreased *p*-Parkin, PINK1, and LC3 protein levels in mice subjected to CLP.

### 3.5. Improved Macrophage Mitochondrial Function Ameliorated IL-27-Induced Hepatic Injury in Mice Subjected to CLP

**Figure 5:**
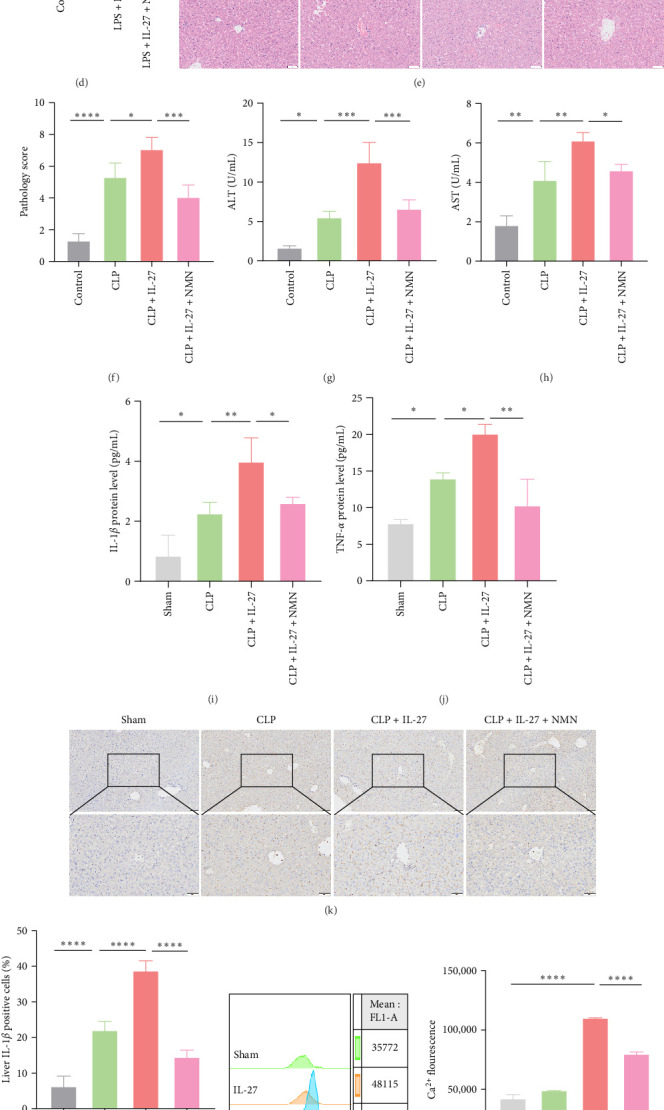
Improved macrophage mitochondrial function ameliorated IL-27-induced hepatic injury in mice subjected to CLP. (A) Changes in ATP content in RAW264.7 cells after treatment with LPS, IL-27 and NMN (*n* = 4; Control vs. LPS + IL-27 *p*  < 0.0001, LPS + IL-27 vs. LPS + IL-27+NMN *p*=0.0119). (B) The protein levels of GSDMD and Caspase 1 in HepG2 cells were determined by western blotting (*n* = 4). (C and D) Statistical analysis of relative GSDMD (*n* = 4; Control vs. LPS *p*=0.0017, LPS vs. LPS + IL-27 *p*  < 0.0001, LPS + IL-27 vs. LPS + IL-27+NMN *p*  < 0.0001) and Caspase 1 (*n* = 3; Control vs. LPS *p*=0.0006, LPS vs. LPS + IL-27 *p*=0.0030, LPS + IL-27 vs. LPS + IL-27+NMN *p*=0.0009) protein expression levels. (E) Mouse hepatic pathological injury was detected by H&E staining (*n* = 4). (F) The pathology determined from H&E staining (*n* = 4; Control vs. CLP *p*  < 0.0001, CLP vs. CLP + IL-27 *p*=0.0378, CLP + IL-27 vs. CLP + IL-27+NMN *p*=0.0008). (G and H) Serum ALT (*n* = 4; Control vs. CLP *p*=0.0204, CLP vs. CLP + IL-27 *p*=0.0002, CLP + IL-27 vs. CLP + IL-27+NMN *p*=0.0009) and AST (*n* = 4; Control vs. CLP *p*=0.0012, CLP vs. CLP + IL-27 *p*=0.0036, CLP + IL-27 vs. CLP + IL-27+NMN *p*=0.0248) levels. (I and J) The concentrations of inflammatory cytokines IL-1*β* (*n* = 4; Control vs. CLP *p*=0.0250, CLP vs. CLP + IL-27 *p*=0.0066, CLP + IL-27 vs. CLP + IL-27+NMN *p*=0.0275) and TNF-*α* (*n* = 4; Control vs. CLP *p*=0.0274, CLP vs. CLP + IL-27 *p*=0.0269, CLP + IL-27 vs. CLP + IL-27+NMN *p*=0.0018) in mouse serum was measured using ELISA. (K and L) The expression of IL-1*β* in liver tissue was detected by immunohistochemistry (*n* = 4; Control vs. CLP *p*  < 0.0001, CLP vs. CLP + IL-27 *p*  < 0.0001, CLP + IL-27 vs. CLP + IL-27+NMN *p*  < 0.0001). (M and N) The Ca^2+^ concentration in RAW264.7 cells were measured by flow cytometry (*n* = 4; Control vs. LPS *p*  < 0.0001, LPS vs. LPS + IL-27 *p*  < 0.0001). (O and P) *Atp2a3* (*n* = 4; Control vs. LPS *p*  < 0.0001, LPS vs. LPS + IL-27 *p*  < 0.0152) and *Camk2d* (*n* = 4; Control vs. LPS *p*=0.0004, LPS vs. LPS + IL-27 *p*=0.0016) mRNA levels in RAW264.7 cells were detected by qPCR. All images were randomly obtained at 40× and 100× visual fields. *⁣*^*∗*^*p* < 0.05, *⁣*^*∗∗*^*p* < 0.01, *⁣*^*∗∗∗*^*p* < 0.001, *⁣*^*∗∗∗∗*^*p* < 0.0001. ATP, adenosine triphosphate; CLP, cecal ligation and puncture; ELISA, enzyme-linked immunosorbent assay; IL, interleukin; LPS, lipopolysaccharide; NMN, nicotinamide mononucleotide; PCR, polymerase chain reaction.

NMN was used to enhance the mitochondrial function of macrophages, and its effect on cocultured HepG2 cells was evaluated. We first explored the effect of NMN on the ATP content of macrophages. IL-27 treatment further increased the ATP content in RAW264.7 cells increased by LPS treatment. However, this effect was reversed by NMN treatment (Figure 5A; *n* = 4). Subsequently, we assessed the effects of changes in the mitochondrial function of macrophages on pyroptosis in cocultured HepG2 cells (Figure 5B; *n* = 4). When NMN was administered along with LPS and IL-27, the protein levels of cleaved GSDMD and cleaved Caspases 1 in HepG2 cells were restored to levels comparable to those in the control groups (Figure 5B–D; *n* = 4). We sought to further clarify the effect of NMN on CLP-induced AHI following IL-27 treatment in vivo. More serious pathological liver injury was observed in the IL-27 treatment group than in the CLP group. However, NMN improved the liver pathology scores (Figure 5E,F; *n* = 4). We then determined the serum ALT and AST levels. Despite an obvious increase in the levels of ALT and AST induced by IL-27 in the CLP model, NMN treatments inhibited this change to some degree (Figure 5G,H; *n* = 4). The changes in serum inflammatory factors levels of IL-1*β* and TNF-*α* were also consistent with this finding (Figure 5I,J; *n* = 4). Consistent with the ELISA results, NMN treatment decreased the hepatic levels of IL-1*β* under IL-27 stimulation in mice subjected to CLP (Figure 5K,L; *n* = 4). Finally, we investigated the potential mechanisms by which IL-27 influences the phagocytic and bactericidal activities of macrophages through the modulation of mitochondrial function. We evaluated the intracellular Ca^2+^ concentrations in macrophages using flow cytometry. The results indicated that LPS treatment upregulated of Ca^2+^ levels in macrophages. However, this effect was attenuated in the presence of IL-27 (Figure 5M,N; *n* = 4). Moreover, *Atp2a3* and *Camk2d* mRNA levels were significantly upregulated after IL-27 treatment (Figure 5O,P; *n* = 4).

## 4. Discussion

The latest definition of sepsis describes it as a clinical syndrome in which the host response to infection is dysregulated, and life-threatening organ dysfunction occurs [22]. Despite advances in our understanding of and treatment of sepsis, it still poses a significant healthcare burden globally. Sepsis-induced AHI can cause hypoxic hepatitis, cholestasis, and impaired protein synthesis [23]. The pathogenesis of liver dysfunction in the context of sepsis is complex and includes excessive inflammatory responses, microcirculation disorders, hepatocyte and liver dysfunction, oxygen free radicals, and lipid peroxidation [23]. Although the use of broad-spectrum antibiotics and supportive therapy improves survival in patients with sepsis, mortality in patients with sepsis and liver dysfunction or failure is still as high as 54%–68% [24].

Excessive inflammatory responses are the main pathophysiological mechanism underlying AHI induced by sepsis [24]. In sepsis-associated AHI, mononuclear or macrophage-derived IL-6 and IL-1 are over-released, leading to liver microcirculation failure, mitochondrial dysfunction, and hepatocyte injury [25]. Therefore, immunotherapy is an important development direction for the molecular treatment of sepsis-associated AHI. Previous studies have confirmed that IL-6 signaling deficiency specifically eliminates microtubule-mediated ERK transport and weakens the killing of bacterial and fungal pathogens by macrophages [26]. IL-27, a member of the IL-6/IL-12 family, plays a complex role in abnormal immune inflammation that occurs during sepsis [11]. A meta-analysis showed that IL-27 is a diagnostic biomarker of sepsis [27]. Our previous data from patients with sepsis-associated AHI also suggested that IL-27 is elevated and promotes hepatic damage and inflammation [15]. Moreover, the knockdown of IL-27R protein expression suppressed hepatic pathological injury and pyroptosis (Figure 1) in the CLP model, highlighting its therapeutic relevance. An important study showed that inflammatory mediators (released by Kupffer cells or circulatory cells) affected hepatocytes, and this suppressive role (in hepatospecific functions and enzymes) has been previously used in cell-based treatments, such as hepatocyte transplantation approaches [28]. In the present study, we further investigated the effects of IL-27 on sepsis-associated AHI from the perspective of bacterial clearance by macrophages.

Macrophages, which are key immunoregulatory cells that kill pathogens, can effectively enhance pathogen clearance and prevent organ damage caused by excessive immune and inflammatory responses [29]. Therefore, enhancing the phagocytic and killing functions of macrophages and reducing the release of inflammatory factors can effectively defend against pathogen invasion, reduce the occurrence of sepsis, and improve prognosis. Previous studies have shown that anti-PD1 therapy can enhance the bacterial clearance rate of Kupffer cells and reduce AHI in the context of sepsis [30]. Thus, our data provide novel insights into the cytokine-driven regulation of macrophage function during sepsis-associated AHI. Previous studies have suggested that IL-27 inhibits the antimicrobial response of neutrophils and macrophages [31, 32]. Specifically, IL-27 modulates neutrophils and macrophage activation via an unknown mechanism. What are the mechanisms underlying this phenomenon?

We then established a model of CLP-induced AHI in WT and IL-27R^−/−^ mice to explore whether IL-27 affected bacterial clearance. IL-27 significantly impaired bacterial clearance from the blood and liver of mice subjected to CLP (Figure 2A,B). Furthermore, macrophage phagocytosis and killing experiments demonstrated that IL-27 inhibited the LPS-induced phagocytosis and killing functions of macrophages (Figure 2C,D). As phagocytes engulf microorganisms, they produce bactericidal ROS that kill the microorganisms [33]. We detected the ROS levels in macrophages using flow cytometry and found that IL-27 reduced LPS-induced ROS levels (Figure 2E–G), which was consistent with the previously reported effect of IL-27 on ROS levels in neutrophils [34].

Mitochondria, as the central hub of cellular metabolism and intracellular signaling, have been implicated in a variety of diseases, including sepsis [35]. The important role of mitochondrial function in macrophage killing has been well documented. Macrophages alter mitochondrial energy substrate utilization, resulting in increased mass, a maximal oxygen consumption rate, and spare respiratory capacity, which permits phagocytosis during sepsis [36]. Trigger receptor 2 (TREM2) enhances bacterial clearance by controlling ROS production during sepsis [37]. However, it is unknown whether IL-27 affects the antimicrobial ability of macrophages by regulating mitochondrial function. In our animal and cell models, we confirmed that the significant impairment of macrophage antimicrobial ability was accompanied by mitochondrial dysfunction after IL-27 treatment (Figure 3). Moreover, impaired mitochondria can induce mitophagy. Recent studies have demonstrated that severe infections are accompanied by mitochondrial dysfunction and abnormal mitophagy [38]. Our cellular and animal studies illustrated that IL-27 further promoted mitophagy (Figure 4), which is consistent with the finding that mitophagy inhibition enhances macrophage activation and antibacterial defense during sepsis [39]. Finally, the pathological damage of the liver was significantly improved after the improvement of mitochondrial function by NMN (Figure 5). Several pharmacological compounds, including coenzyme Q10, mitoquinone, resveratrol, and NMN, have been used to enhance mitochondrial function. Coenzyme Q10, mitoquinone, and resveratrol primarily act as antioxidants that protect mitochondria from oxidative stress [40]. NMN is an essential NAD^+^ precursor that improves mitochondrial function by regulating cellular NAD^+^ levels. A previous study reported that NMN can prevent mitochondrial dysfunction and alleviate multiorgan failure via SIRT3 signaling in mice with sepsis [20]. Moreover, another study demonstrated that NMN supplementation protects the aging liver from oxidative-stress-induced damage [41]. In our study, macrophage-induced hepatocyte pyroptosis after IL-27 intervention was ameliorated by NMN treatment, and we can currently infer that this effect of IL-27 should be realized partially dependent on aggravating the impairment of macrophage mitochondrial function. However, the potential pharmacological targets (Coenzyme Q10, mitoquinone, and resveratrol) and molecular mechanisms by which IL-27 affects sepsis-associated AHI by altering macrophage mitochondrial function require more solid support from our further studies in the future.

Finally, we explored the potential mechanisms by which IL-27 influences the phagocytic and bactericidal activities of macrophages through the modulation of mitochondrial function. It is widely recognized that mitochondrial dysfunction disrupts the homeostatic regulation of cellular Ca^2+^ levels [42, 43]. Calcium channel blockers reduce the uptake of *Escherichia coli* bacteria by peritoneal macrophages in mice [44]. Ca^2+^ signaling pathways activate macrophages via their involvement in ROS production [45]. Moreover, Ca^2+^ plays a crucial role in macrophage activation by facilitating actin rearrangement, NADPH oxidase activation, and protein kinase C (PKC) stimulation [46, 47]. Our results indicated that IL-27 reduced LPS-induced Ca^2+^ levels in macrophages, and this was accompanied by the upregulation of *Atp2a3* and *Camk2d* mRNA levels (Figure 5M–P). Therefore, the decrease in Ca^2+^ levels resulting from mitochondrial dysfunction may constitute a significant factor contributing to the impairment of macrophage phagocytosis and killing activity induced by IL-27.

This study raised some issues that deserve attention. ATP production and the consumption of ATP are important prerequisites for macrophage function. In our study, the ATP content of RAW264.7 was detected after treatment with LPS and IL-27. Unexpectedly, compared with LPS treatment alone, combined treatment with LPS and IL-27 further increased the ATP content. However, this was contrary to the results of mitochondrial function, which suggested that IL-27 further impaired LPS-induced mitochondrial dysfunction (based on COX protein levels and JC-1 staining). Because ATP levels are related to ATP production and utilization, our results showed a strong imbalance between ATP production and ATP utilization, which also explains the obstacle of energy utilization in macrophages from another perspective. Whether this is because of macrophage dysfunction, leading to impaired ATP utilization, needs to be demonstrated experimentally.

In summary, IL-27 aggravates liver damage and increases systemic inflammatory response by inhibiting bacterial clearance by macrophages. This mechanism may be related to the aggravation of mitochondrial dysfunction by IL-27, which subsequently leads to a decrease in intracellular Ca^2+^ levels, impairing the antimicrobial function of macrophages.

## 5. Conclusion

IL-27 aggravates AHI in sepsis by impairing the bacterial phagocytosis capacity of macrophages via aggravating mitochondrial dysfunction.

## Data Availability

The data related to this article may be shared by the corresponding author upon reasonable request.
